# Early Focal Segmental Glomerulosclerosis as a Cause of Renal Allograft Primary Nonfunction

**DOI:** 10.1155/2013/565697

**Published:** 2013-05-23

**Authors:** Emma J. Griffin, Peter C. Thomson, David Kipgen, Marc Clancy, Conal Daly

**Affiliations:** ^1^University of Glasgow Medical School, Glasgow G12 8QQ, UK; ^2^Renal Unit, Western Infirmary, Dumbarton Road, Glasgow G11 6NT, UK; ^3^Department of Pathology, Southern General Hospital, Glasgow G51 4TF, UK

## Abstract

*Background.* Primary focal segmental glomerulosclerosis (FSGS) is one of the commonest causes of glomerular disease and if left untreated will often progress to established renal failure. In many cases the best treatment option is renal transplantation; however primary FSGS may rapidly recur in renal allografts and may contribute to delayed graft function. We present a case of primary nonfunction in a renal allograft due to biopsy-proven FSGS. *Case Report.* A 32-year-old man presented with serum albumin of 22 g/L, proteinuria quantified at 12 g/L, and marked peripheral oedema. Renal biopsy demonstrated tip-variant FSGS. Despite treatment, the patient developed progressive renal dysfunction and was commenced on haemodialysis. Cadaveric renal transplantation was undertaken; however this was complicated by primary nonfunction. Renal biopsies failed to demonstrate evidence of acute rejection but did demonstrate clear evidence of FSGS. The patient was treated to no avail. *Discussion.* Primary renal allograft nonfunction following transplantation is often due to acute kidney injury or acute rejection. Recurrent FSGS is recognised as a phenomenon that drives allograft dysfunction but is not traditionally associated with primary nonfunction. This case highlights FSGS as a potentially aggressive process that, once active in the allograft, may prove refractory to targeted treatment. Preemptive therapies in patients deemed to be at high risk of recurrent disease may be appropriate and should be considered.

## 1. Background

A well-recognised cause of renal allograft dysfunction is recurrence of the primary glomerular disease. Primary focal glomerulosclerosis (FSGS) is one of the commonest causes of glomerular disease [1], and in those who have subsequently undergone successful transplantation, its contribution to progressive renal allograft dysfunction and eventual allograft loss is well described [2]. The clinical picture of recurrent primary FSGS usually takes two main forms: early graft dysfunction which is characterised by the emergence of significant proteinuria through the graft within the first few weeks following transplantation [3] with subsequent loss of excretory function, and late graft dysfunction where there is a progressive rise in urinary protein excretion and serum creatinine many months or years following transplantation. 

We describe a case where biopsy has proven that early recurrence of FSGS is the cause of primary nonfunction in the renal allograft.

## 2. Case Report

A 32-year-old man presented to renal services in early 2009 with a 3-week history of facial puffiness. He was found to have nephrotic syndrome with an initial serum albumin of 22 g/L, proteinuria quantified at 12 g/L, and marked peripheral oedema. He proceeded to renal biopsy which demonstrated focal segmental glomerulosclerosis with no evidence of immune complex deposition, only mild to moderate chronic arteriopathy, and minimal chronic tubulointerstitial damage. Whilst his renal excretory function was well preserved at presentation, he experienced a relatively rapid subsequent decline in function in the context of persisting nephrotic syndrome despite treatment with both high-dose prednisolone and ciclosporin. He commenced haemodialysis nine months after his initial presentation.

In July 2011 a kidney from a 27-year-old donor who died following an intracerebral haemorrhage became available. The kidney provided a 1 HLA A, 1 HLA B, and 0 HLA DR mismatch and negative T cell and B cell cross-matches. Successful surgical transplantation of the kidney occurred following a total cold ischaemic time of 8.5 hours and anastomosis time of 31 minutes. The patient received alemtuzumab (CAMPATH) induction followed by low-dose tacrolimus (target trough levels 5–7 ng/mL) and mycophenolic acid (Myfortic) 360 mg twice daily. Cotrimoxazole 480 mg prophylaxis was given, and as both donor and recipient were noted to be CMV antibody positive, valganciclovir prophylaxis was commenced. The EBV status of both donor and recipient was negative.

Over the ensuing days there was persistent proteinuria of 18 g/L prior to the patient becoming oligoanuric and required regular haemodialysis. Throughout the perioperative and postoperative periods he remained haemodynamically stable with a proteinuria of 7.1 g/L and minimal urine output. Serial ultrasonography of the allograft consistently demonstrated no evidence of hydronephrosis and adequate perfusion. On postoperative days 4, 5, and 6 he received daily 500 mg intravenous methylprednisolone on the presumption that the cause of delayed graft function was acute rejection, proceeding to renal allograft biopsy on day 6. This demonstrated mild to moderate flattening and loss of tubular epithelial cell cytoplasm consistent with expected acute ischaemic injury related to transplantation. There was also moderate arterial intimal oedematous thickening. There was no arteritis and no evidence of other cell mediated or antibody mediated rejection. Immunoperoxidase staining for C4d was negative in glomerular and peritubular capillaries. There were no glomerular abnormalities and no chronic tubulointerstitial damage.

He remained oligoanuric and a subsequent biopsy on day 15 showed similar features with moderate acute tubular injury and minimal chronic inflammation involving less than 5% of the tubulointerstitium. In this biopsy the arterial intimal oedema had decreased; again there was no arteritis and no other features diagnostic of cell mediated or antibody mediated acute rejection. Immunoperoxidase staining for C4d remained negative.

**Figure 1:**
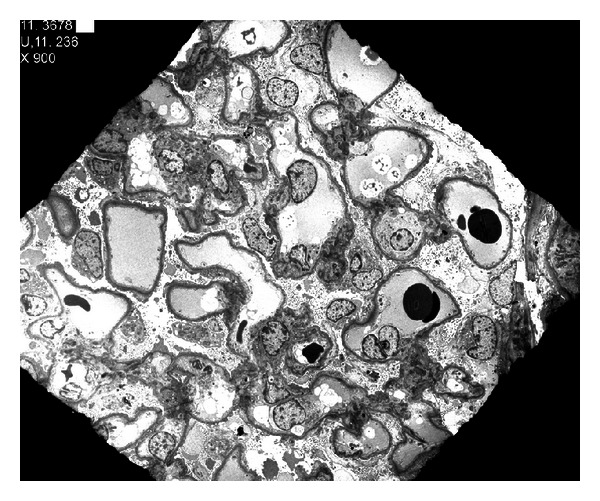
Electron micrograph (×900).

**Figure 2:**
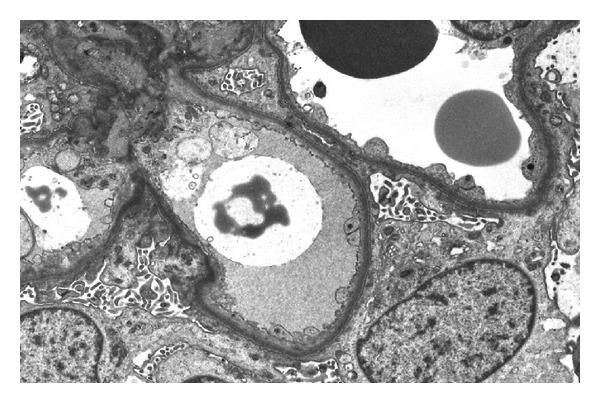
Electron micrograph (×1100). These EM appearances were consistent with early recurrent FSGS. The presence of neutrophils in tubules raised a possibility of ascending bacterial urinary tract infection.

The clinical picture persisted and a transplant biopsy on day 35 demonstrated neutrophil polymorph casts in about 15% of tubules, together with predominantly acute inflammation in about 15% of the interstitium. Apart from this, light microscopy was very similar to the previous biopsy, with moderate features of acute tubular injury. There was no evidence of cell mediated or antibody mediated rejection. Staining for C4d was negative. On immunofluorescence, no significant staining was seen. Electron microscopy revealed widespread visceral epithelial cell (podocyte) foot process effacement, involving more than 90% of the glomerulus examined (Figures 1 and 2). There was mild patchy glomerular basement membrane folding. No endothelial cell tubuloreticular inclusions and no electron dense deposits were seen. The mesangium appeared normal. The general consensus was a recurrence of FSGS causing delayed graft function.

A 3-week course of alternate day plasma exchange was undertaken in conjunction with oral prednisolone and continued tacrolimus and mycophenolic acid. These interventions were of little effect and the patient remained anuric and continued his dependency on regular haemodialysis.

Six months following transplantation the patient attended otolaryngology with recurrent sore throat. CT of his head/neck was performed reported as “bilateral ulcerating lesions of both tonsils with excess parapharyngeal tissue and small cervical lymph nodes.” Bilateral tonsillectomy was performed with pathology demonstrating an Epstein Barr Virus (EBV) driven posttransplant diffuse large B cell lymphoma involving both tonsils.

No CNS or marrow involvement of the disease was found on imaging. Ann-Arbour stage was 2AE and the patient has since undergone R-CHOP chemotherapy followed by field radiotherapy. He continues on haemodialysis 3x/week and will not be reactivated on the transplant list until his lymphoma has been successfully treated and there is no subsequent evidence of recurrent disease for a minimum of 5-year period.

## 3. Discussion

Primary FSGS with rapid renal decline is not uncommon and in many patients renal function continues to deteriorate despite treatment with steroids and immunosuppressants [4]. Studies have shown that if FSGS is left untreated, it will often follow a progressive course to established renal failure and in only about 10% of cases there will be spontaneously complete remission [5]. Previously, prognosis for FSGS was poor despite treatment; currently with longer courses of treatment prognosis has been improved significantly [6]. Factors contributing to a less favorable outcome include the degree of proteinuria, histological findings, and response to therapy [7]. Our patient had a poor renal prognosis from the outset with persistent proteinuria that proved refractory to treatment. In such patients renal transplantation is considered the treatment of choice [8]; however recurrent FSGS is always a concern.

The pathogenesis of recurrent FSGS is still not completely understood however. In animal models early onset recurrence has been associated with a circulating permeability factor thought to be released from abnormal clonal T cells [9]. This factor significantly increases permeability to albumin and is thought to cause podocyte injury classifying FSGS as a podocyte disease. A bioassay is now available to quantify the glomerular permeability factor in patients prior to renal transplant. Although higher levels are associated with the development of recurrent FSGS, the predictive value of an abnormal test result is limited [9] and this is not used routinely to identify “at risk” patients who are proceeding to transplantation. Another circulating factor that has recently been identified is the soluble form of the urokinase receptor (suPAR) which has been shown to activate podocyte *β*_3_ integrin which can lead to recurrent FSGS pathology. In two thirds of patients presenting with FSGS suPAR levels were found to be elevated [10].

Recurrent FSGS following transplantation has been shown to develop in up to 30% of renal transplant patients [11]. Factors which influence the risk of recurrence include a second transplant after loss from recurrence, childhood transplantation, rapid progression to uraemia, and being of white ethnicity [12, 13]. Neither posttransplant duration of dialysis nor choice of immunosuppression has any influence over graft survival or recurrence of FSGS. Early recurrence is more common and presents with massive proteinuria hours to days following transplant. Graft biopsies at first can appear normal on light microscopy but show effacement of foot processes on electron microscopy. After some time biopsies show a more “cellular” quality which includes foam cell accumulation, endocapillary proliferation, and thickened podocytes, in the later stages and these then appear as sclerosing lesions [14]. Late recurrence develops over months to years following transplantation and presents similarly to early recurrence. Outcomes are much the same but late recurrence is insidious compared with early recurrence.

Following transplantation our patient developed primary nonfunction with initial renal transplant biopsy appearances consistent demonstrating acute tubular injury. The transplant biopsy on day 35 demonstrated evidence of recurrent FSGS on electron microscopy which appears to have compounded the overall renal injury to this transplant and ultimately led to primary nonfunction and a lack of renal recovery following tubular injury. This case highlights FSGS as a potentially aggressive process that, once active in the allograft, may subsequently prove refractory to targeted treatment. This raises the question of whether Preemptive therapies in patients deemed to be at high risk of recurrent disease may be appropriate and should be considered. Current practice has been based on the role of plasmapheresis or immunoadsorption with protein A in conjunction with steroid and calcineurin inhibition. Sustained beneficial effects have been reported with the use of the chimeric monoclonal anti-CD20 antibody Rituximab although this has not always been successful [15, 16]. Whether Preemptive treatment with plasmapheresis and/or anti-CD20 antibodies could offer an improved likelihood of successful transplantation in such patients remains unproven [17].

Our patient developed posttransplant lymphoproliferative disease (PTLD) 6 months after transplantation which pathology reported as a diffuse large B cell lymphoma. PTLD is an increasingly important complication following transplantation as it is unpredictable, can lead to graft loss, and is at times fatal [18]. The majority of cases are associated with Epstein Barr Virus (EBV) which drives tumour formation in B cells and are a consequence of the negative effects of immunosuppressants given following solid organ transplantation and the decreased T cell surveillance which alters the body's control of EBV [19]. Clinical presentation is extremely varied and includes fever, lymphadenopathy, and gastrointestinal symptoms. Most common sites for involvement are lymph nodes (59%), liver (31%), lung (29%), and kidneys (25%) with tonsillar presentation being down at 10% [20]. Increased incidence has been noted to occur with more intense immunosuppression. To our knowledge, the recurrence of FSGS following transplantation does not increase the chances of developing PTLD and both are thought to be mutually exclusive. 

When considering subsequent transplantation in patients who have previously lost an allograft through recurrent FSGS, outcomes remain poor. A retrospective paediatric study of 29 patients who received a graft for FSGS was conducted; 3 received a second transplant for recurrent FSGS. Of these, all 3 experienced severe proteinuria and had biopsy proven recurrent FSGS [21]. Other studies have quoted figures of over 80% for the development of FSGS following previous transplant and previous recurrence [22, 23]. We therefore suggest that in such cases targeted Preemptive therapies may have a role and should be considered.
